# Spinal mechanisms of neuropathic pain: Is there a P2X4-BDNF controversy?

**DOI:** 10.1016/j.ynpai.2017.04.001

**Published:** 2017-04-08

**Authors:** Marzia Malcangio

**Affiliations:** Wolfson Centre for Age Related Diseases, IoPPN, King’s College London, United Kingdom

## Abstract

More than a decade ago the novel concept that glial cells are major players in the modulation of pain mechanisms in the spinal cord has started a prolific series of work addressing the modalities of neuron-glia communication. Mike Salter with Kazuhide Inoue laboratories introduced ATP as pivotal mediator for such communication via activation of P_2_X_4_ receptors expressed by microglia in the dorsal horn ipsilateral to a peripheral nerve injury. Activation of P_2_X_4_ receptors result in release of the neurotrophin BDNF, which, through the activation of neuronal TrkB receptors, alters neuronal excitability and this effect is associated with behavioural ipsilateral allodynia. This viewpoint article compares the evidence supporting a biological relevance of the P_2_X_4_ and BDNF system in neuropathic pain with recent data which question such importance. Having read this article, readers will be able to formulate their own opinion on such controversy.

## Highlights

• This viewpoint article compares the evidence supporting a biological relevance of the P_2_X_4_ and BDNF system in neuropathic pain with recent data which question such importance.

## Introduction

More than a decade ago the seminal work of Linda Watkins and Joyce DeLeo groups started a novel line of research in the pain field, promoting the novel concept that glial cells are major players in the modulation of pain mechanisms in the spinal cord (Watkins and Maier, 2003, DeLeo and Yezierski, 2001). Following damage to peripheral nerves or tissues, microglia in the dorsal horns can detect the increased activity of primary afferent fibres and dorsal horn neurons that results from the damage. Microglia alter their morphology, proliferate and exert pro-nociceptive actions. An intriguing question raised in such a scenario was how do neurons communicate to the microglia? With the aim of answering this, the pioneering work by Catherine Abbadie’s laboratory at Merck and Mike Salter with Kazuhide Inoue introduced the chemokine CCL_2_ and extracellular ATP as pivotal mediators for such communication via activation of the CCR_2_ and P_2_X_4_ receptors, respectively (Abbadie et al., 2003, Coull et al., 2005). This viewpoint is concerned with the neuron-microglia pathway that starts with ATP activation of P_2_X_4_ receptors on microglia. Activation of this receptor results in release of the neurotrophin BDNF, which, through the activation of neuronal TrkB receptors, alters neuronal excitability and this effect is associated with behavioural hyperalgesia and allodynia under neuropathic conditions. Recent data have challenged the presence of both P_2_X_4_ and BDNF in spinal cord microglia thereby sparkling stimulating and healthy scientific debates, which are summarised here. Rather than follow the sequence of the signalling pathway which has been covered in several review articles, here BDNF will be introduced and discussed before the P2X4 receptors. Indeed BDNF is the critical pro-nociceptive factor which mediates microglia-neuron communication under neuropathic conditions whilst under normal conditions BDNF is expressed in neurons.

### The neurotrophins

The neurotrophin family of trophic factors includes nerve growth factor (NGF), brain-derived neurotrophic factor (BDNF), neurotrophin 3 (NT3) and neurotrophin 4/5 (NT4/5). The neurotrophins are initially synthesised as preforms of about 30 kDa in size, prior to their expression as mature forms of about 13 kDa. The cellular actions of the mature neurotrophins are mediated by two cell surface receptors. Whilst all of the neurotrophins can activate the low affinity receptor p75 and promote apoptosis via JNK (stress-activated protein kinases (SAPK)/Jun amino-terminal kinases) and nuclear factor–κB (NFκB) pathways, activation via higher affinity tropomyosine receptor kinases (Trk) are more specific. NGF and NT-3 for example, signal via the TrkA receptor whilst BDNF and NT4/5 activate the TrkB receptor and NT-3 signals via the TrkC receptor. Trk receptors are tyrosine kinase receptors which dimerise following ligand binding. Phosphorylation of several residues leads to activation of intracellular pathways including ras-raf MAP kinase, PLC and PI3 kinase (Chao, 2003, Pezet and McMahon, 2006).

The neurotrophins play critical roles during the development of the nervous system and promote both survival and differentiation of central and peripheral neurons (Mitre et al., 2017).

### BDNF and acute pain transmission

In adulthood BDNF via an action on TrkB receptors can regulate synaptic strength both in the hippocampal CA1 region that is involved with learning and memory, and at the first sensory synapse in the dorsal horn of the spinal cord, which is the first relay station of noxious signalling from the periphery (Malcangio and Lessmann, 2003, Ji et al., 2003)

Sensory neurons that express BDNF constitutively belong to the peptidergic population of neurons-with small cell bodies in the dorsal root ganglia (DRG). Mature BDNF (14 kDa) is stored in large dense core vesicles (LDCVs), which also contain substance P (SP) and calcitonin gene-related peptide (CGRP). BDNF-expressing sensory neurons are pseudounipolar as their axons project to both the periphery of the body and centrally to the dorsal horn of the spinal cord (Michael et al., 1999). The peripheral axon terminals of neurons that express BDNF respond to noxious stimuli and are therefore classified as nociceptors. Their central axons project to the superficial laminae of the dorsal horn of the spinal cord and contain LDCVs that are anterogradely transported from the cell bodies. More than twenty years ago, we observed that the release of BDNF from the central terminals of nociceptors occurs in an activity-dependent fashion after electrical stimulation of the dorsal roots using short bursts of high intensity and high frequency pulses (Lever et al., 2001). Therefore, we suggested that certain noxious stimuli that produce bursting activity in nociceptive fibres, result in the release of BDNF, which binds TrkB receptors that are discretely expressed by dorsal horn neurons (Mannion et al., 1999, Salio et al., 2005). Dimerization and phosphorylation of TrkB receptors leads to activation of the intracellular Ras pathway and auto-phosphorylation of ERK kinase which is followed by post-translational modifications such as phosphorylation and transcription. The activation of TrkB in dorsal horn neurons results in phosphorylation of the NR1 subunit of N-methyl-D-aspartate (NMDA) receptor for glutamate and augmentation of glutamate-NMDA receptor mediated excitation (Kerr et al., 1999, Slack et al., 2004). This effect of BDNF infers a pro-nociceptive role of endogenous BDNF that is best revealed under conditions of chronic pain in which BDNF plays a significant role (Pezet et al., 2002).

### BDNF under chronic pain conditions

BDNF expression in DRG neurons displays a plasticity that is dependent of the availability of NGF in the periphery. The expression level of BDNF in nociceptive neurons that also express TrkA receptors is up-regulated after systemic NGF treatment and in peripheral inflammatory conditions in concomitance to increased NGF levels (Kerr et al., 1999). Under both conditions, activity-induced release of BDNF in the dorsal horn is increased and BDNF potentiates glutamate-mediated neuronal excitation driven by NMDA receptors (Lever et al., 2001). In a behavioural scenario, the sequestration of spinal cord BDNF by intrathecal administration of a neutralising TrkB receptor antibody, reduces formalin- and carrageenan-pain related behaviours (Kerr et al., 1999, Thompson et al., 1999) indicating a pro-nociceptive role of BDNF. However, the use of a TrkB antibody does not provide a definite answer as it may also sequester NT4, which for instance, can potentiate morphine-induced analgesia at least in the brain stem (Lucas et al., 2003). Thus, the development of conditional nociceptive neuron-BDNF null mice (nociceptor-specific Cre strain; Nav1.8-Cre) (Zhao et al., 2006) provides a neat model to evaluate the role of sensory neuron-derived BDNF in chronic inflammatory and neuropathic pain.

**Fig. 1:**
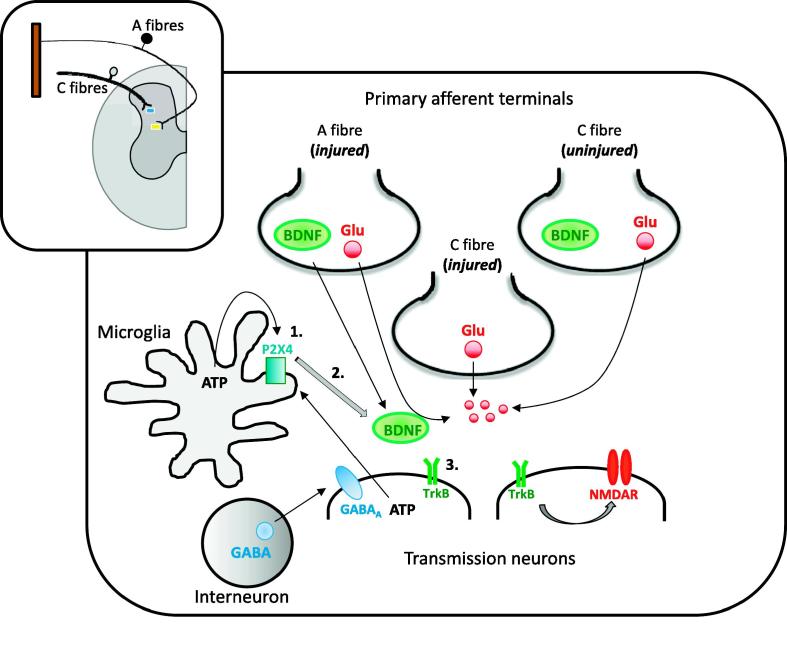
The ATP-P2X4-BDNF pathway in the spinal cord. Following peripheral nerve injury, BDNF expression is down-regulated in injured C fibres and unchanged in uninjured C fibres, and BDNF is *de novo* expressed by A fibres. The overall result of these changes in expression is that neuron-derived BDNF release in the dorsal horn is reduced compared to normal conditions. Instead BDNF is released by microglia (2) through ATP activation of the P_2_X_4_ receptor (1). BDNF activation of TrkB receptors (3) results in NMDA receptor activation and downregulation of potassium-chloride co-transporter KCC2 in dorsal horn neurons and causes an intracellular accumulation of chloride ions. When such neurons express GABA_A_ receptors, GABA activation, rather than hyperpolarization, induces depolarization.

Evidence obtained in nociceptive neuron-BDNF null mice supports a pro-nociceptive role of BDNF in inflammatory pain as in these mice both formalin and carrageenan-induced pain behaviours are significantly impaired (Zhao et al., 2006). However, in nerve injury models of neuropathic pain, these mice do not display alterations in the development of pain-like behaviour (Zhao et al., 2006) suggesting no major role of BDNF derived from small sensory-neurons under neuropathic conditions. Consistent with a limited role of BDNF in pain-like behaviour under neuropathic conditions is the observation that the expression of BDNF is down-regulated in damaged nociceptive neurons following peripheral nerve injury (Michael et al., 1999). Furthermore, under nerve-injury conditions, the release of BDNF after activation of primary afferent fibres is dramatically impaired in the dorsal horn (Lever et al., 2003), even though the residual BDNF can still potentiate NMDA receptor activity in primary afferent terminals (Chen et al., 2014) (Fig. 1).

Interestingly, the neuronal shortage of BDNF is compensated by the microglia, which store BDNF in vesicles and release BDNF in response to increases in both primary afferent barrage and dorsal horn neuron activity when neuronal release of BDNF is impaired by a peripheral nerve injury. The release of BDNF from microglia is mediated by ATP activation of P_2_X_4_ receptors (Fig. 1). Indeed, evidence from Salters’ and Inoue’s laboratories indicates that following peripheral nerve injury, microglia in the ipsilateral dorsal horn of the spinal cord, *de novo* express P_2_X_4_ receptors via increased expression of interferon regulatory factor 8 (IRF8) and IRF5 which bind to the promoter region of the p2x4 gene (Tsuda et al., 2003, Coull et al., 2005, Tsuda, 2016). Notably, IRF5 regulates P_2_X_4_ expression in a gender bias fashion- inducing expression exclusively in males (Sorge et al., 2015). The binding of extracellular ATP to P_2_X_4_ promotes the release of BDNF by microglia in a biphasic fashion, consisting of an early (5 min) and delayed (60 min) component which both depend on extracellular calcium (Trang et al., 2009). The release of BDNF by microglia is mediated by activation of p38 MAP kinase and SNARE (soluble N-ethylmaleimide-sensitive factor attachment protein receptor)-mediated exocytosis (Trang et al., 2009). Once released, BDNF activation of TrkB receptors can result in significant downregulation of potassium-chloride co-transporter KCC2 in dorsal horn neurons, especially those in lamina I. This mechanism causes an intracellular accumulation of chloride ions and consequently, if such neurons express GABA_A_ receptors, GABA activation, rather than hyperpolarization, induces depolarization (Coull et al., 2005). The overall result of an activation of the P_2_X_4_-BDNF-GABA_A_ pathway is a contribution to the reduced inhibition and increased excitation in the dorsal horn, especially in lamina I and in male mice, under neuropathic conditions. Relevantly, compounds that are selective KCC2 activators and reduce intracellular chloride ions concentration, produce a significant anti-allodynic effect in neuropathic animals (Gagnon et al., 2013).

The impact of microglia-derived BDNF on chronic pain states is highlighted by the observation that genetic depletion of BDNF from microglia (BDNF deleted in a tamoxifen-manner in CX_3_CR_1_ positive cells) results in failure to develop neuropathic allodynia in male mice. Intriguingly, in female mice, whilst microglia respond with increased activity to increased peripheral afferent fibre barrage, they are not critical for the development of allodynia as female mice may use adaptive immune cells instead of the microglia (Sorge et al., 2015). Although an intriguing suggestion, the contingent of T cells is small in the spinal cord even one-two weeks after nerve injury. Future studies on the gender issue will clarify the role of distinct immune cell subtypes in neuropathic pain.

### P2X4-BDNF pathway in microglia

After a number of years from its discovery, recent evidence has been inconsistent in supporting a P_2_X_4_-BDNF pathway in microglia. For instance, microglia isolated from the dorsal horns of nerve-injured mice do not display a significant presence of the BDNF gene whilst they show upregulation of other microglial genes such as csfr1, cx3cr1 and ctss1 which are implicated in neuropathic pain development (Denk et al., 2016). Furthermore, colony-stimulating factor-1 receptor (CSF-1R) signalling in microglia is P_2_X_4_-receptor independent whereas cx3cr1, ctss1 and indeed bdnf can be regulated by CSF1-R (Guan et al., 2016). Colony stimulating factor 1 (CSF1) is a secreted cytokine which is *de novo* expressed by injured primary afferent fibres (Guan et al., 2016) similarly to the chemokines CCL_2_ and CCL_21_ which are also expressed by DRG neurons after injury (Thacker et al., 2009, Dansereau et al., 2008, Jung et al., 2008, Biber et al., 2011). However, microglia do not express CCR_2_ receptors (Jung et al., 2009) and whilst they express CCR_7_ and CXCR_3_ receptors for CCL_21_, these receptors are not critical for the development of neuropathic allodynia (Biber et al., 2011). Therefore amongst these cyto(chemo)kine systems, the microglial CSF1R and downstream adapter protein DAP12 (12 kDa transmembrane protein) are better suited to mediate sensory neuron-microglia communication of pain signalling after nerve injury. The fact that they do not control P_2_X_4_ expression suggests the existence of discrete pathways for activation of genes in the microglia.

Controversial data stimulate scientific discussion and challenge the *status quo*. When considering the P_2_X_4_-BDNF pathway in microglia in another model system such as the hippocampus, evidence supports a role for microglial BDNF in synaptic plasticity. Data indicate that hippocampal microglia produce and secrete BDNF and that the removal of BDNF from microglia (BDNF deleted in a tamoxifen-manner in CX_3_CR_1_ positive cells), whilst not altering the total level of BDNF protein, is associated with a decrease of phosphorylated TrkB receptor in synaptosomes obtained from brain tissue (Parkhurst et al., 2013). Furthermore, the culture media of P_2_X_4_-activated microglia can phosphorylate TrkB receptors in synaptosomes and provides indirect evidence for biological activity of BDNF constitutively released by microglia (Parkhurst et al., 2013). In a behavioural context specific to hippocampus, mice lacking microglial BDNF show a reduction in performance improvement after motor training and reduced learning-induced synaptic formation in hippocampus compared to controls (Parkhurst et al., 2003). Thus microglia express and release BDNF which has functional consequences and regulates hippocampal plasticity. Whether this phenomenon is gender specific remains to be ascertained. Nevertheless, the hippocampal results reinforce the strength of the fact that a functional P_2_X_4_-BDNF relationship exists *in vivo*.

### P_2_X_4_ receptors

The P_2_X receptors belong to a family of calcium-permeable non-selective cation channels gated by extracellular ATP (North, 2002). The P_2_X_7_ receptor for instance, is expressed by immune cells such as macrophages and microglia and mediates the release of the cytokine interleukin-1β in the dorsal horn of the spinal cord (Clark et al., 2010a). Microglial P_2_X_7_ receptors play critical roles in mediating IL-1 β pro-nociceptive actions in the spinal cord (Clark et al., 2010a).

The P_2_X_4_ receptors are highly calcium permeable and expressed by microglia at very low levels (Tsuda et al., 2003) in the intracellular lysosomal compartment. We know that in macrophages the P_2_X_4_ receptors rapidly traffic to the plasma membrane following endolysosomal secretion in response to phagocytosis (ingestion of zymosan particles) (Stokes and Surprenant, 2009). In contrast, classical activation of macrophages by lipopolysaccharide/interferon-γ (LPS)/IFNγ) results in a dramatic reduction of both ATP-activated membrane currents and P_2_X_4_ membrane expression in macrophages. Thus, regulation in macrophages reveals that the receptor becomes functional in response to initial phagocytic stimuli but returns to a non-functional state during sustained activation of macrophages. The suggestion is that instead of P_2_X_4_, it is the P_2_X_7_ receptor that takes the leading role in sustained activation of macrophages (Stokes and Surprenant, 2009). Therefore, if the trafficking of P_2_X_4_ receptors in microglia were comparable to macrophages, then we could speculate that the P_2_X_4_ receptor mediates microglial mechanisms in the initial phases of neuropathic pain induction whilst P_2_X_7_ receptors become involved in maintenance. P_2_X_7_ receptor activation mediates the release of the cytokine IL-1 β and the lysosomal enzyme cathepsin S from microglia *via* a p38 MAP kinase pathway (Clark et al., 2010b). Whether BDNF can be released following P_2_X_7_ receptor activation remains an intriguingly possibility to be investigated.

In the context of neuropathic pain, an increased concentration of extracellular ATP would be responsible for P_2_X_4_ and P_2_X_7_ receptors recruitment. The source of ATP would be neurons, microglia and especially astrocytes, as indeed astrocytes release ATP in a glucocorticoid-dependent manner and circadian fashion (Masuda et al., 2016, Koyanagi et al., 2016). Specifically, adrenalectomised mice do not develop allodynia following nerve injury and systemic administration of glucocorticoid to adrenalectomised and nerve injured mice, induces ipsilateral mechanical allodynia. In the spinal cord, glucocorticoids enhance glucocorticoid regulated SKG-1 (AGC kinase family) expression in spinal astrocytes and the release of ATP through pannexin-1 hemichannels. Notably, the microglial receptor involved in the glucocorticoid diurnal effect on microglia has been identified as the P2Y_12_ excluding an involvement of the P_2_X_4_ receptor (Koyanagi et al., 2016).

Consistent with an early role of the P_2_X_4_ receptor in neuropathic pain is the observation that P_2_X_4_ k/o mice develop less severe allodynia soon after peripheral nerve injury compared to wild type controls. However, neuropathic allodynia in P_2_X_4_ k/o mice remains less severe than in wild type for up to 4 weeks after injury suggesting a role in the maintenance of pain (Ulmann et al., 2008). Likewise, P_2_X_7_ k/o mice develop less severe allodynia as soon as 3 days after nerve injury and display reduced allodynia compared to wild type for several weeks (Chessell et al., 2005).

Therefore, the behavioural data in transgenic mice do not help to establish a temporal sequence for functional relevance of P_2_X_4_ and P_2_X_7_ receptors in neuropathic pain mechanisms. Other means of investigation would have to be devised if pre-clinical data were to offer guidance to drug discovery and clinical trials. For instance, single nucleotide polymorphisms (SNPs) in the p2x7 gene that are known cause gain-of pain-function and loss of-pain-function result in changes in i) pore and protein levels in recombinant cell studies and ii) pain sensitivity in diabetic patients (Ursu et al., 2014).

## Conclusion

This view point has laid out some of the current evidence in favour and against the microglial P2X4-BDNF pathway playing a significant role in neuropathic pain with the aim to provide an unbiased opinion. There is a need for novel targets and therapeutic strategies for neuropathic pain treatment. BDNF plays a pro-nociceptive role in chronic pain and under neuropathic pain conditions the P_2_X_4_–BDNF functional relationship *in vivo* may provide future analgesic therapies, such as P_2_X_4_ antagonists and KCC2 enhancers. However, some studies have questioned the presence of BDNF in spinal cord microglia and others have suggested that P_2_X_4_ receptor-mediated mechanisms may apply to specific types of neuropathic pain. The delineation of pathways other than the P_2_X_4_-BDNF, offers valid alternative to harness neuron-non neuronal communication pathways for the discovery of innovative therapies.

## Acknowledgements

I wish to thank Dr Karli Montague for scientific discussion and for producing Fig. 1. Research in MM lab is currently supported by the Arthritis Research UK (grant number 20020), Medical Research Council (MR/M023893/1) European commission FP7/2007-2013 under grant agreement 602133 and under grant agreement grant 603191.

## Conflict of interest

The authors declare that they have no conflicts of interest.
